# In Situ Growth of Mushroom‐Shaped Adhesive Structures on Flat/Curved Surfaces via Electrical Modulation

**DOI:** 10.1002/advs.202408680

**Published:** 2024-11-05

**Authors:** Hongmiao Tian, Yingze Li, Duorui Wang, Qi Chen, Yuanze Jiang, Tianci Liu, Xiangming Li, Chunhui Wang, Xiaoliang Chen, Jinyou Shao

**Affiliations:** ^1^ Micro‐and Nano‐technology Research Center State Key Laboratory for Manufacturing Systems Engineering Xi'an Jiaotong University Xi'an Shaanxi 710049 China; ^2^ Frontier Institute of Science and Technology (FIST) Xi'an Jiaotong University Xi'an Shaanxi 710049 China

**Keywords:** curved surface, dry adhesive, electrical modulation, in situ growth

## Abstract

Gecko‐inspired adhesives have an extraordinary impact on robotic manipulation and locomotion. However, achieving excellent adhesive performance on curved surfaces, especially undevelopable surfaces, is still challenging. This can be attributed to a considerable difference between the fabrication method and practical necessity, i.e., the adhesive structures are generally fabricated on a flat substrate whereas the manipulating surface is curved, resulting in a low adhesive strength. Here, an in‐situ growth strategy is proposed to fabricate mushroom‐shaped structures at micro/nano‐scale via electrical modulation on flat or curved surfaces. Since the adhesive structures are directly grown on target surfaces without a transfer procedure, they exhibit a large contact area and stress uniformity at the interface, corresponding to an excellent adhesive force. A comparison between grown structures using the proposed method and those fabricated using traditional approaches suggests that the adhesive forces are identical for flat testing surfaces, while the difference can be up to 4 times for developable surfaces and even 25 times for undevelopable surfaces. The proposed adhesion strategy extends the application prospects of gecko‐inspired adhesives from flat surfaces to curved ones, composed of developable and undevelopable surfaces, opening a new avenue to develop gecko‐inspired adhesive‐based devices and systems.

## Graphical abstract

Geometrical distortions during the transfer process limit the adhesion strength of gecko‐inspired adhesives on curved surfaces, especially undevelopable surfaces. A method capable of growing mushroom‐shaped adhesion structures in situ on surfaces with different curvatures is realized by applying a modulated electric field. Mushroom‐shaped adhesive materials fabricated by this method exhibit superior adhesion strength on curved surfaces than all similar structures reported in recent years.

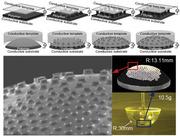

## 1 Introduction

Geckos could climb on vertical walls and horizontal ceilings due to their excellent attachment characteristics via micro/nano‐structures on the surfaces.^[^ ^1^, ^2^, ^3^, ^4^ ^]^ The Van der Waals interactions provide a sufficient load to adhere to the target surfaces without morphology and materials consideration of target surfaces, demonstrating extensive applications in space operation, industrial manufacturing, and many other fields.^[^ ^5^, ^6^, ^7^, ^8^, ^9^, ^10^, ^11^, ^12^, ^13^, ^14^, ^15^ ^]^ Until now, gecko‐inspired adhesive structures have attracted tremendous attention, accompanied by significant progress in material design,^[^ ^16^, ^17^, ^18^, ^19^, ^20^, ^21^, ^22^ ^]^ fabrication methods,^[^ ^23^, ^24^, ^25^ ^]^ gripping/releasing strategy,^[^ ^10^, ^26^, ^27^, ^28^, ^29^, ^30^, ^31^, ^32^ ^]^ and so forth. Notably, micro/nano‐scale mushroom‐shaped morphology endows superior adhesive strength than others (such as flat, spherical, concave, and spatula morphology), because the mushroom‐like cap can eliminate the stress singularity at the contacting interface, stabilize defects at the plate–substrate interface and control cracks appear first in the center region at separation.^[^ ^33^, ^34^, ^35^, ^36^ ^]^ However, mushroom‐shaped adhesive structures are generally fabricated on flat substrates,^[^ ^37^, ^38^, ^39^, ^40^ ^]^ exhibiting low adhesion on curved surfaces, restricting their applications on curved surfaces, such as robotic fingers and special‐shaped optical elements. Achieving good adhesive performance on curved surfaces, especially for undevelopable curved ones, is still challenging.

To obtain adhesive structures on curved surfaces, the micro/nano‐structures can be fabricated on a flat substrate first and then transferred to the curved surface. By methods such as single‐molecule modification, it is possible to realize the transfer of the fabricated structure from the substrate with near‐zero adhesion, thereby ensuring that the micro/nanostructures are transferred from the fabricated substrate without wrinkles, cracks, or defects.^[^ ^41^ ^]^ However, when combined with a curved surface, the mushroom‐like structure may be deformed due to the change of state from flat to curved.^[^ ^42^ ^]^ Shape distortion would require extraordinary considerations in shape mapping and geometric design, inducing numerous constraints on fabricating methods. Especially for undevelopable surfaces (such as spherical surfaces), it is scarcely possible to directly transfer the adhesive structures from a flat film to a curved one. With the development of conformal manufacturing techniques, many methods have been developed to fabricate micro‐ and nanostructures directly on 3D curved surfaces, such as additive manufacturing, laser machining, and imprint lithography, which may create desired structures on curved surfaces.^[^ ^43^, ^44^, ^45^, ^46^, ^47^, ^48^ ^]^ However, it is challenging to generate mushroom‐shaped morphology at micro/nano‐scale with a large area. Therefore, developing a novel approach to fabricated mushroom‐shaped adhesive structures on curved surfaces or even undevelopable surfaces is necessary to promote the development of gecko‐inspired adhesives.

In practice, a gecko's toe surface is typically curved with clumps of setae with highly split‐formed nanoscale ends, which exhibit high aspect ratios and therefore higher flexibility, beneficial for reducing the contact stress concentration between their toes and the target surfaces and thus improving the contact area. However, fabricating polymers into highly split structures with a high aspect ratio similar to gecko setae is extremely difficult. In detail, the properties of existing materials cannot rival those of biological tissues, which are subject to collapse or entanglement with each other as being processed into highly split structures with high aspect ratio,^[^ ^49^ ^]^ resulting in the reduction of the effectiveness of their adaptation to curved surfaces. Therefore, fabricating adhesive structures with morphology matching the target substrate directly on curved surfaces seems to be an alternative strategy to achieve strong adhesion on the curved target surfaces.

Despite the biological structures generated on a curved surface, they are tightly bound during curvature changes in the gecko toe surface, resulting in a stable attachment of geckos on various walls. Notably, there is an apparent difference between the artificial and biological adhesive structures in the forming mechanism. The artificial adhesive structures can be obtained by mechanical fabrication and then transferred to the target surface,^[^ ^50^, ^51^ ^]^ usually needing a variety of equipment and complex procedures, as well as easily inducing geometrical distortion and poor combination. In contrast, the biological adhesive structures on the gecko's toes are in situ grown via cell proliferation, from nothing to micro/nano‐structures.^[^ ^52^, ^53^ ^]^ Moreover, these biological structures are formed in one step. When the in situ growth of biological adhesive structures is introduced to the formation of mushroom‐shaped adhesive structures, new ideas may be produced on the challenge of micro/nano‐structures formed on curved surfaces without geometrical distortion.

**Figure 1:**
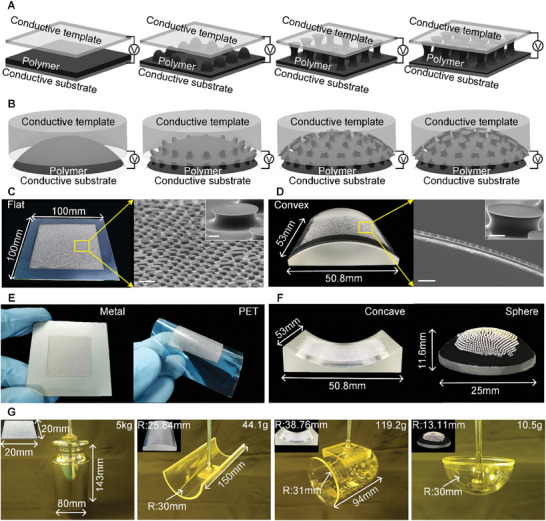
Schematic illustration of the in situ growth of mushroom‐shaped adhesive structures on numerous target surfaces. A,B) Schematic of mushroom‐shaped structures electrically grown on flat and curved surfaces, respectively. C) Mushroom‐shaped adhesive structures grown on flat surfaces with 100 × 100 mm areas. The scale bars in the SEM image and inset are 400 and 60 µm, respectively. D) Grown mushroom‐shaped adhesive structures on the concave surface with a 50.8 × 53 mm area. The scale bars are 400 and 100 µm, respectively. E) Adhesive structures grown on various substrates such as metal and PET. F) Adhesive structures grown on curved surfaces with different geometry, consisting of concave and spherical surfaces. G) Grasping ability of grown structures on different target surfaces, namely a weight as a flat surface of 5 kg, a convex plate as a concave surface of R = 30 mm, a beaker as a convex surface of R = 31 mm, and a globe holder as a spherical surface of R = 30 mm.

Herein, we propose an in situ growth of mushroom‐shaped adhesive structures on polymer film on flat or curved surfaces under the actuation of an electric field on the fluidic polymer (**Figures** **1**A,B). Owing to the favorable modulation of the electric field modulated by the use of an upper electrode plate with a matching morphology to the surface of the supporting substrate the electrical distributions on the polymeric film have no apparent difference between the flat and curved surfaces, thus leading to structure growth on flat/curved surfaces without geometrical distortion. Initially, a polymer film is produced on the target surfaces. When an electric field is applied to the polymer film, the electrostatic force generated at the air‐polymer interface drives the polymer upward to the upper electrode, forming the periodic “mushroom stem.” At first glance, the forming process is similar to that of the electrohydrodynamic patterning method, which both drive the polymer to form periodic structures.^[^ ^54^, ^55^ ^]^ In practice, to guarantee the polymer grows into a mushroom‐shaped structure with good adhesion, an electric wetting force is introduced via a dielectric layer on the bottom surface of the upper electrode to drive the polymer to spread on the contacting surface, resulting in the “mushroom tip”, which is seldom considered by the conventional electrohydrodynamic patterning approach. Since the micro/nano‐structures are grown from a polymer film to a mushroom‐shaped geometry, the process is performed in situ without extra transfer. Therefore, the adhesive structures are fabricated on the target surfaces (flat, curved, and undevelopable surfaces), which is difficult to achieve by conventional approaches.

## 2 Results and Discussion

**Figures** **1**C,D demonstrate the mushroom‐shaped grown structures on flat (100 × 100 mm) and convex surfaces (53 × 50.8 mm). The micro/nano‐scale mushroom‐shaped morphology with a large area is generated on the target surfaces. The electrostatic force on the polymer film determines the structural geometry, and the electrode size adjusts the forming area. Notably, the electrostatic force is incurred on the polymer film without a strict requirement on the target surface conductivity, achieving the electrical in situ growth of mushroom‐shaped structures on target surfaces with or without conductivity (Figure 1E). For instance, the conductive metal can be directly used as the electrode, creating mushroom‐shaped structures on its surface; the non‐conducting polyethylene glycol terephthalate (PET) film can be attached to another electrode to introduce an electric field, also resulting in mushroom‐shaped structures on a PET film. Generally, the polymer exhibits low electrical conductivity, comparable to deionized water the generated electrostatic force is mainly determined by the material permittivity. Thus, various materials can be used to create mushroom‐shaped structures. Considering that elastomeric materials are beneficial for increasing the flexibility of the structure to conform on the contact surface, polydimethylsiloxane (PDMS), a polymer widely used on gecko‐inspired adhesives, is adopted to fabricate the mushroom‐shaped adhesive structures. Other elastic materials can also be used for adhesive structures, such as Norland optical adhesive (NOA), polyurethane (PU), and polyurethane acrylate (PUA), which can all be obtained by the proposed in situ growth method (Figure S1, Supporting Information), reflecting the universality of the proposed approach on forming materials. The mushroom‐shaped structures can be fabricated on a developable surface (concave as an example) or an undevelopable surface (sphere as an example) with a well‐modulated electric field, as shown in Figure 1F. The structures obtained by electrically in situ growth demonstrate superior adhesive performance on manipulating surfaces of various shapes (Figure 1G); for example, adhesive structure on a flat surface for gripping a weight of 5 kg, adhesive structure on a convex surface (R = 25.84 mm) for engaging a concave plate with R = 30 mm and weight of 44.1 g, adhesive structure on a concave surface (R = 38.76 mm) for gripping a beaker with R = 31 mm and weight of 119.2 g, and adhesive structure on a spherical surface (R = 13.11 mm) for engaging a globe holder with R = 30 mm and weight of 10.5 g. The gripping performance of these adhesive structures on flat or curved surfaces highlights the necessity of shape‐matching between adhesive and testing surfaces. Therefore, fabricating adhesive structures on flat or curved surfaces without geometrical distortion is crucial.

### 2.1 Electrical In Situ Growing Mechanism of Adhesive Structures on Target Surfaces

**Figure 2:**
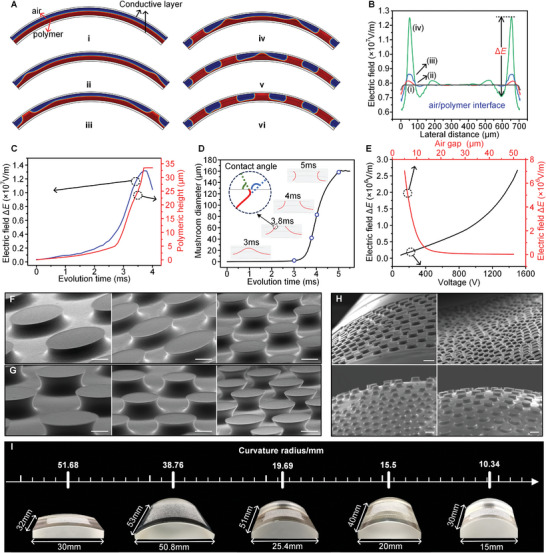
Analysis of the mushroom‐shaped adhesive structures grown under an electric field. A) Numerical simulations obtained the dynamic evolution of polymer film under an external electric field. Here, blue and red colors represent air and polymer, respectively. B) Electric field distribution at the air–polymer interfaces corresponding to different stages (i–iv) in A. C) Variation in electric field Δ*E* and polymer height versus evolution time. D) Evolution of mushroom tip diameter with the growing time and the change in contact angle on three‐phase boundaries. The macroscopic contact angle and the microscopic contact angle are marked in blue and green in the inset at 3.8 ms, respectively. E) Influence of applied voltage and air gap between the upper electrode and the polymer surface on the electric field Δ*E*. Grown adhesive structures on F) curved surfaces (R = 38.76 mm) with different voltages and G) varying gaps of air. The scale bars are 100 µm. H) Grown adhesive structures on convex surface (R = 10.34 mm), concave surface (R = 31.01 mm). and spherical surface (R = 13.11 mm). The scale bars are 400 µm. I) Grown adhesive structures on a convex cylinder with a curvature radius of 51.68, 38.76, 19.69, 15.5, and 10.34 mm, respectively.

To understand the in situ growth mechanism on target surfaces under an electric field, a numerical model is proposed to describe the fluidic behavior based on the Gauss equation to represent the electric field, the Navier‐Stokes equation for the flow field, and the Cahn‐Hilliard to express the fluidic air and polymer. The details of the numerical model are provided in Section 1 (Supporting Information). **Figure** **2**A demonstrates the dynamic evolution of polymer film on a curved surface (a convex surface is used as an example) with the dynamic motion shown in Movie S1 (Supporting Information). According to the variation in polymer morphology, the fabrication process can be distinguished into stage I, namely the vertical growth stage to form the mushroom stem, and stage II, namely the horizontally increasing stage for generating a mushroom tip. Initially, the polymeric film is coated on the convex surface via natural leveling controlled by gravity and surface tension (Figure 2A‐i). When the electric field is applied to the polymer film, the electrostatic force generated at the air‐polymer interface drives the polymer to overcome the surface tension and viscous force, moving upward to the opposite electrode (Figure 2A‐ii), corresponding to stage I. When the polymer contacts the upper electrode, the electrowetting force generated at the three‐phase boundary promotes the polymer dispersion on the contacting surface (Figure 2A‐iii), corresponding to stage II. Subsequently, the mushroom‐shaped structures are generated from the electrode edge to the center region (Figures 2A‐iv–vi), attributed to the initial distribution of electrostatic force, further determined by the electric field (Figure S2, Supporting Information). Thus, it is reasonable to depict the polymeric evolution through the electrical distribution of the polymer film.

At stage I of vertical growth, the polymer moves upward to produce the mushroom stem, and the growing behavior is explored from the electric field at the air‐polymer interface (Figure 2B). Owing to the non‐uniformity of the electric field near the electrode edges, the film becomes mechanically unstable in this area, and the pillars proliferate. Once the polymer starts to flow upward near the electrode edge, the film near the template edges tends to flow toward the electrode edges to supply the polymer. Therefore, the periodic growth of pillars is initialized at the electrode edge, which continues toward the center. It is worth noting that the mushroom structures are not formed one by one from the outside to the inside in the sequence of the inner one beginning to flow after the outer one is finished. In practice, the polymer in the adjacent region starts to flow just before the outer polymer generates the mushroom‐shape geometry, which leads to a competition for polymer between the structures in the neighboring regions, i.e., the volume of polymer available for each structure is limited. To describe the evolution of polymer film, we defined the following parameter Δ*E* = *E*_max_ − *E*_min_, the effective electric intensity, and corresponds to the effective driving force. Δ*E* increases as the polymer moves higher, illustrated by the curves in Figures 2B‐i–iv. Furthermore, the variation in Δ*E* versus polymer height at stage I is described in Figure 2C. The electric field and the pillars' height exhibit positive feedback to one another, i.e., the stronger electric field pulls the polymer to a more significant height, and the polymer at a more considerable height induces a stronger electric field. This mutual positive feedback effect keeps the pillars growing until they reach the bottom surface of the upper electrode.

At stage II of horizontal growth, the polymer spreads at the bottom surface of the upper electrode, forming the mushroom tip under the electrowetting force. Figure 2D displays that the mushroom changes with the spreading evolution. The mushroom diameter increases and then reaches a constant value with the polymer moving along the electrode surface. During the mushroom tip formation process, the contact angle between the fluidic polymer and the bottom surface of the upper electrode changes from an obtuse to an acute angle, defined as the macroscopic contact angle (marked by blue in the inset of Figure 2D). However, the actual contact angle is always smaller than 90°, defined as the microscopic contact angle (marked by green in the inset of Figure 2D). Obviously, there is a twist exists near the location where the polymer edge contacts the upper electrode (inset in Figure 2D). Here, the macroscopic contact angle is obtuse when the polymer starts to contact the upper electrode under the coupling effect of electrostatic force and surface tension. In addition, owing to the presence of a dielectric layer on the bottom surface of the upper electrode, the electrowetting effect keeps the microscopic contact angle between the polymer and the upper electrode always less than 90°. With the evolution of the polymer morphology under the electric field, the macroscopic contact angle is gradually transformed into an acute angle, equaling the microscopic contact angle finally. When the polymer contacts the upper electrode, Δ*E* decreases because the medium connecting the electrode pair is a polymer (high dielectric constant) rather than air (low dielectric constant), i.e., the electrostatic force becomes small. It is conducive to obtaining an equilibrium state between the electrostatic force, the electrowetting force, and the friction force, thus the polymer does not continue to spread horizontally. The above factors result in the polymer not growing indefinitely, and the process continues cyclically, resulting in the mushroom‐like structure growing from the outside to the inside, eventually forming a periodic structure with similar dimensions. In addition, since the entire process occurs in a short period of time and the polymer is curing immediately after the formation of the periodic mushroom‐shaped structure, the structure that forms first and the one that forms later have uniform sizes and periods. In practice, since the growth behavior of the structure, i.e., the polymeric flow, is driven by the electrostatic force, it is necessary to control the uniform distribution of the initial electric field intensity at the air‐polymer interface, so that the mushroom‐shaped structures of uniform size and period can be obtained on the substrate surface (Figure S17, Supporting Information). For this purpose, an upper electrode plate matching the morphology of the substrate surface is required to control the spacing between the upper and lower electrode plates to achieve a uniform electrical distribution between the electrode plates.

The effective electric intensity, Δ*E*, is a crucial factor in determining the fluidic behavior of polymer, modulated by experimental parameters, such as applied voltage and air gap between the upper electrode and the polymer surface (Figure 2E). Δ*E* gradually increases with the increase in voltage, i.e., the applied voltage enhances the driving force. Additionally, Δ*E* sharply decreases with the increase in air gap; a small air gap benefits the electrostatic force to promote the polymeric flow. Thus, modulating the grown structures by adjusting experimental parameters is possible. The effect of applied voltage on the mushroom‐shaped structure's growth is depicted in Figure 2F, with the voltage of 200, 300, and 500 V, respectively, on a convex target surface with curvature of 38.76 mm (Figure S3, Supporting Information). A large voltage decreases the periodicity (the distance between the centers of structures). It increases the aspect ratio (the value of polymeric height to diameter) by adjusting the effective electric intensity Δ*E*. Similarly, the formed adhesive structures are influenced by the air gap between the upper electrode and polymer surface with values of 75, 55, and 35 µm, respectively (Figure 2G). A small air gap can produce structures with a small periodicity and a large aspect ratio. The finite element analysis of the polymer growth process on the convex surface with different parameters is shown in Figure S11 (Supporting Information). Obviously, adhesive structures with smaller periods and sizes can be obtained with the decrease of the air gap and the increase of the voltage, respectively (corresponding to the increase of the electric intensity). The impact of voltage and air gap on the mushroom‐shaped structure growth again suggests that electric intensity Δ*E* is the dominant factor in the growing process, and a larger Δ*E* is beneficial for lowering the periodicity and enhancing the aspect ratio, and vice versa. In addition, by further decreasing the air gap to 100 nm in the finite element analysis, arrays of mushroom‐shaped structures of tens of nanometers were obtained (Figure S19, Supporting Information), demonstrating the potential for fabricating adhesive structures in nanoscale through in situ growth strategy. Furthermore, except for the mushroom‐shaped geometry, other geometries can be obtained by adjusting the magnitude of electrostatic force. For instance, if the electrostatic force (determined by Δ*E*) is not sufficient to drive the polymer to conquer viscous force and surface tension to move upward, the morphology of micro‐lens can be obtained (Figure S18A, Supporting Information); if the electrostatic force cannot drive the polymer to move along the bottom surface of the upper electrode after contacting each other, the geometry of pillars can be obtained (Figure S18B, Supporting Information).

Figure 2H demonstrates the mushroom‐shaped structures growth on the convex surface (R = 10.34 mm), concave surface (R = 31.01 mm), and the top and bottom region of the spherical surface (R = 13.11 mm), accompanied by the actual images shown in Figure S4, Supporting Information. The mushroom‐shaped structures at the micro‐scale are perpendicular to the curved surface without noticeable geometry distortion, conducive to maintaining good adhesive performance. The mushroom‐shaped structures are electrically grown in numerous curved surfaces with curvature radii of 51.68, 38.76, 19.69, 15.5, and 10.34 mm (Figure 2I). The curvature of these surfaces is precisely controlled by different cylindrical lenses, along with the complementary lenses as the opposite electrode. There is no strict restriction on the curvature of the target surface due to the flexibility of the structure grown on the target surface via an electric field. The mushroom‐shaped structures can be well fabricated on flat, curved surfaces of different curvatures. The electrically growing strategy is a versatile and straightforward fabricating method to produce mushroom‐shaped structures at micro/nano‐scale on flat or curved surfaces without obvious geometrical distortions, which conventional methods, such as lithography, 3D printing, transfer technique, etc., cannot achieve.

### 2.2 Adhesion Enhancement Mechanism of Grown Structures on the Curved Surface

**Figure 3:**
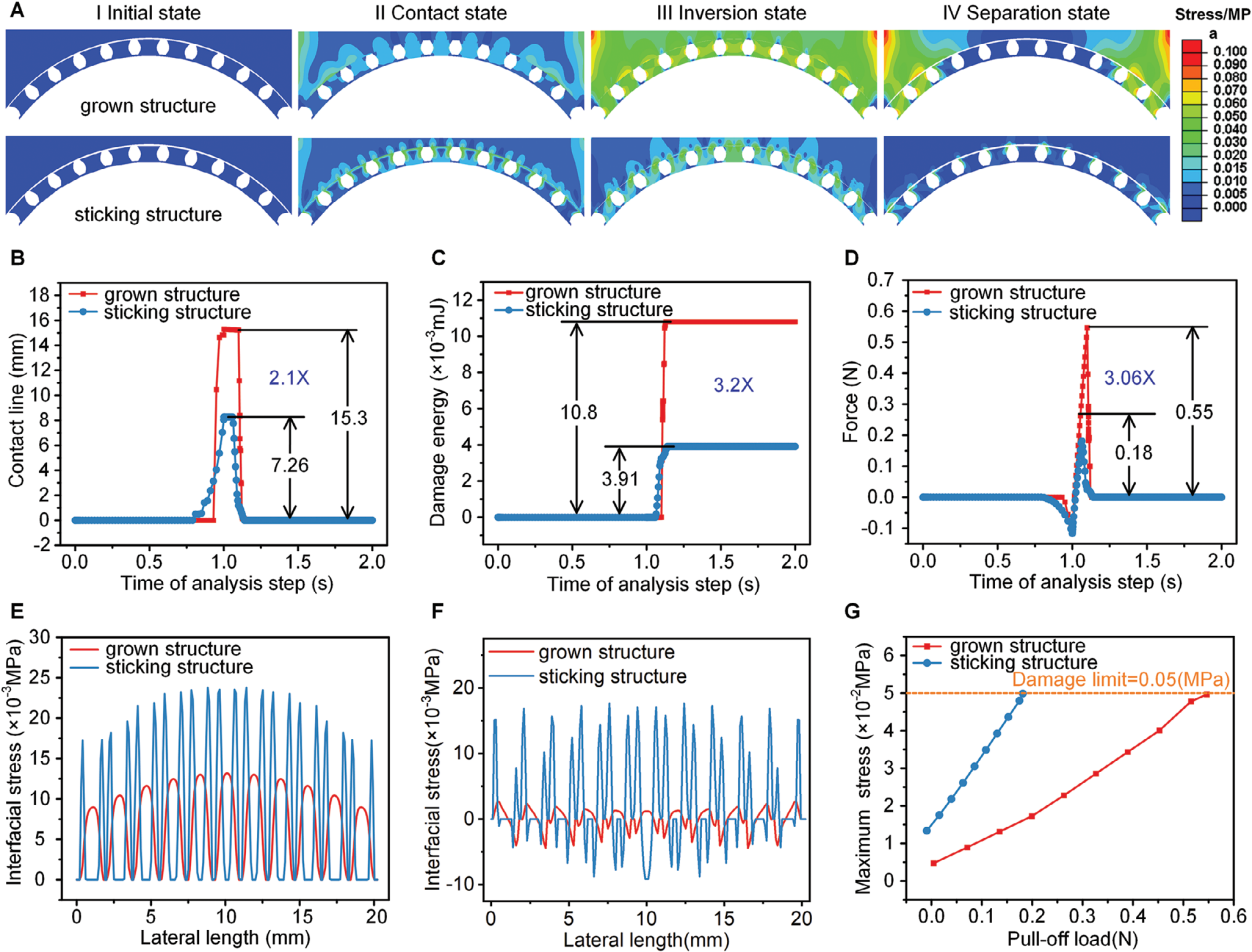
Adhesion enhancement mechanism of the grown structures on curved surfaces. A) Dynamic behavior of the grown and sticking structures on curved surfaces at (I) initial state, (II) contact stage, (III) inversion state, and (IV) separation state; cloud atlas representing the internal stress. B) Evolution of the contact line for different adhesive structures as a function of process time. C) Evolution of the damage energy for different adhesive structures as a function of process time. D) Adhesive force as a function of process time for different adhesive structures. E,F) Stress at the interface between the adhesive and curved surface at contact and inversion states. G) Evolution of interfacial stress acting on the damage limit in the separating procedure.

A numerical model is implemented based on interfacial cohesive zone theory to investigate the adhesion mechanism of mushroom‐shaped structures on the curved surface. This model is applied to analyze the contact–separation process for two scenarios: a grown (adhesive structure directly grown on a curved surface) and a sticking structure (adhesive structures fabricated on a flat surface and then stuck on the curved surface). The details are provided in Section 2 (Supporting Information). **Figures** **3**A,B illustrate the dynamic behavior of both adhesive structures contacting a curved surface and separating from it, with the cloud atlas representing the stress distribution, comprising the initial stage (there is a gap between two surfaces without contact), contact state (the maximum contact area is achieved), inversion stage (the interfacial stress becomes from compressive stress to tensile stress) and separation stage (the adhesive structures are separated from the target surface). The corresponding dynamic evolutions are presented in Movies S2 and S3 (Supporting Information). Here, the convex surface is used as a substrate, and the complementary concave surface acts as the opposite surface. At first glance, there is no difference between the two cases in the initial stage. However, the topological morphology of the grown structure array's top surface is distinguished from that of the sticking structure array (Figure 3A‐I). The whole topological morphology of the grown structure array is curved due to the electrode pair being used with a curved surface. In contrast, the topological morphology of the sticking structure is numerous broken lines, attributed to the deformation of adhesive structures as sticking to the curved surface. Consequently, the interfacial stress concentration of sticking structures is more evident than grown structures in contact and inversion states (Figures 3A‐II,III). The grown structures are more easily to conformal contact with the curved surface and difficult to separate from the adhered surface, generating a larger adhesion in the separation state (Figure 3A‐IV). Finite element analysis of the contact‐separation behavior of grown structure and sticking structure on concave surfaces with corresponding convex surfaces is shown in Figure S12 (Supporting Information). The contact lines and adhesive forces as a function of the time of analysis step during contact‐separation of grown and sticking structures on convex surfaces in finite element analysis are shown in Figure S13 (Supporting Information). Similarly, the stress concentration of the sticking structure is more pronounced than sticking structure on concave surface, resulting a larger contact area (corresponding to a higher adhesive force).

The superiority of grown structures on curved surfaces is further explored from the contact area (a dominating factor in the contracting process) and damage energy (a dominating factor in the separating process). Figure 3B demonstrates the variation in the contact line during the contacting/separating process, wherein the value of the grown structure is 15.3 mm, and that of the sticking structure is 7.26 mm in the contact state, with a difference of 2.1 times. The contact line differentiation implies that the grown structures exhibit a significant adhesive strength compared to the sticking structure, further attributed to the interfacial stress distribution on the contact stage. Figure 3C illustrates the variation in damage energy for two scenarios during the contacting/separating processes. The damage energy of the grown structure is 10.8 × 10^−3^ mJ, and one of the sticking structures is 3.91 × 10^−3^ mJ. The difference of 3.2 times implies that the grown structure needs much more energy to destroy the interface energy, attributed to a larger adhesion. Based on the contact area and damage energy, the grown structure of 0.55 N and the sticking structure of 0.18 N corresponded to a difference of 3.06 times. The final adhesive force is shown in Figure 3D, highlighting the superiority of grown structures on curved surfaces compared to sticking structures.

Figures 3E,F indicate the interfacial stress distribution on the contacting interface at contact and inversion states, respectively. Usually, when the elements in the numerical model are small enough, the separation stress between the flat pillar and the flat surface will concentrate at the outer edge of the pillar and increase to infinity (Figure S15B, Supporting Information). However, a similar phenomenon does not appear in Figure 3F, even though the element size is already very small (Figure S16, Supporting Information). This can be attributed to the fact that the target surface in the finite element model is curved, and its curvature is not equal to that of the sticking structures’ tips (Figure S9A, Supporting Information), leading to the specificity of the contact‐separation process between them. Specifically, as shown in Figure 3E, when the sticking structures approach the target surface, the interfacial stress concentrates at their outer edges because the curvature of their tips is larger than that of the target surface. In contrast, the curvature of the tips of the grown structures is equal to that of the target surface (Figure S9B, Supporting Information), so the interfacial stress is more uniform. At the initial moment when the sticking structures start to move away from the target surface (Figure S16A, Supporting Information), their center regions first exert separation stress on the target surface, while their edges still exert compressive stress on the target surface because the curvature of their tips is much larger than that of the target surface, which leads to a lower adhesive force (Figure 3D). The grown structures, on the other hand, have the same curvature as the target surface and therefore exhibit a more uniform stress distribution (Figure S16B, Supporting Information), which leads to a higher adhesive force. This matches the evolution of adhesive behavior shown in Figures 3A,B. Also, the action of interfacial stress on the energy damage is explored and presented in Figure 3G. The damage limit is 0.05 MPa, defined as the critical value of the micro‐crack generated at the adhered interface. As the interfacial stress exceeds the damage limit, the crack is produced, promoting the separation behavior at the adhered interface. The sticking structure achieves the limit quickly in the separating process, whereas the grown structure needs more time. This phenomenon implies that the grown adhesive structures are much more challenging to separate from the curved surface, matching the dynamic evolution in Figures 3A,C. The grown adhesive structures exhibit excellent performance compared with the sticking structures based on the variation in the interfacial stress on the contacting/separating process and the damage evolution.

### 2.3 Adhesive Performance of the Grown Structures on Target Surfaces

**Figure 4:**
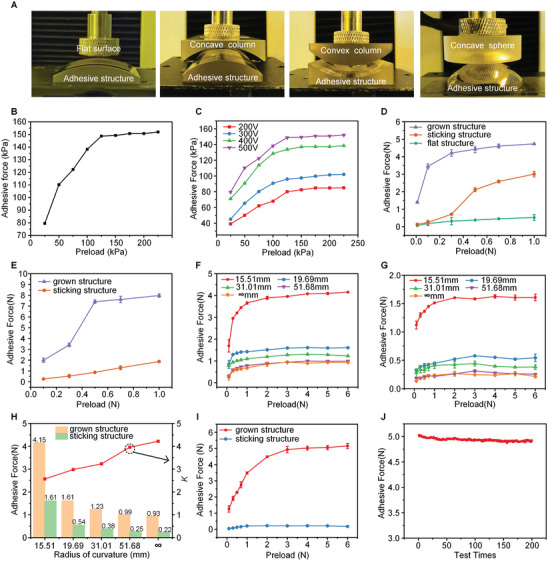
Adhesion of the grown structures on diverse testing surfaces. A) Serial scenes are designed to test the adhesive performance, composed of flat surface versus adhesive structure, concave column versus adhesive structure, convex column versus adhesive structure, and concave sphere versus adhesive structure. B) Variation in the adhesion strength as a function of preload force for the flat testing surface. C) Influence of applied voltage on the adhesive performance of grown structures on flat surfaces. D) Variation in the adhesion strength on convex test surface as a function of preload force for tested samples, grown, sticking, and flat structures. E) Variation in the adhesion strength on concave test surfaces as a function of the preload force for the tested samples, composed of grown and stick structures. F,G) The adhesive performances of the grown and sticking structures on testing surfaces with various curvatures, respectively. H) Comparing the adhesive force of grown and sticking structures to several curvature radii. I) Variation in the adhesion strength as a function of preload force for the testing surface of an undevelopable spherical surface. J) Test cycles of adhesion performance of grown structure on the spherical surface.

To evaluate the adhesive performance of grown structures on target surfaces, three different samples are tested, namely the grown structure on the curved surface (grown structure), the sticking structure on the curved surface (sticking structure), and the adhesive structure on the flat surface (flat structure). Here, unique scenes are designed to test the adhesion performance, comprising testing surfaces of flat surfaces, concave or convex columns and concave spheres, and adhesive structures on complementary surfaces (**Figure** **4**A). First, the adhesive performance of grown structures is explored on flat substrate against the flat testing surface (Figure 4B). The adhesive force increases with the preload to a constant value of 150 kPa. The saturated adhesive strength is nearly identical to that of the adhesive structure obtained by the conventional method (photolithography and molding techniques are adopted here with the fabricated adhesive structure, and its corresponding adhesive force is shown in Figure S5, Supporting Information). This implies that the growth strategy does not sacrifice the adhesive performance despite only one forming step. The influence of applied voltage on the adhesive performance of the grown structure on flat surfaces is shown in Figure 4C, in which the flat surfaces are used as the substrate and the opposite testing surface. The adhesive strength increases as the voltage increases since a larger voltage leads to the grown structure with a dense distribution (the tested grown structures of 200, 300, 400, and 500 V are shown in Figure S6, Supporting Information), suggesting that the forming parameters modulate the adhesive performance. Specifically, the size and period of the structures become smaller as the applied voltage increases, increasing adhesive force. This phenomenon also implies that the optimal size and period of the mushroom‐shaped structures for maximum adhesion strength exist, which has been studied in our previous work.^[^ ^56^ ^]^ Therefore, by varying parameters such as voltage or air gap, it is possible to control the growth of polymer into arrays of mushroom‐shaped structures with optimal size and period, corresponding to a superior adhesion property.

A comparison of three adhesive structures on a curved surface is presented in Figure 4D. The adhesive structure is fabricated on a concave surface (R = 19.69 mm) or flat surface (R = ∞), and the testing surface is convex column surface (R = 19.69 mm). Regardless of preload, the grown structure exhibits a more significant adhesive force than the other two cases, with a sequence of adhesive force from large to small of the grown structure, sticking structure, and flat structure. Especially for a slight preload, the difference exceeds one order of magnitude since the sticking and flat structures cannot conformally contact the curved surface due to the structural distortion on contacting the curved surfaces, wherein the adhesive forces are almost identical for both structures. The adhesive performance is also explored with the adhesive structure fabricated on the convex column surface (R = 19.69 mm) and the testing surface of the concave column surface (R = 19.69 mm), as shown in Figure 4E. Here, the substrate and testing surfaces are used in reverse order to the case in Figure 4D. The flat adhesive structure is not tested since it is impossible to contact a concave testing surface. Similarly, the grown structure demonstrates superior adhesive force than the sticking structure. Here, the difference is more prominent than the convex testing surface because the conformal contact is much more challenging to achieve for the concave testing surface.

Figures 4F,G show the adhesive performances of the grown and sticking structures on testing surfaces with various curvatures, respectively. The adhesive structures are fabricated on convex columns with a 15.51 mm curvature, and the testing surfaces are concave columns with curvature of 15.51, 19.69, 31.01, 51.68, and ∞ mm (i.e., flat surface). For both grown and sticking structures, the case of matching curvature exhibits the most significant adhesive force. With increasing the radius of curvature, the adhesive force decreases continuously. Notably, the adhesive force of grown structures is larger than that of sticking ones, attributed to the structural deformation that occurred in the contacting and separating process (Figure 3). The difference between the grown structure and the sticking one is shown in Figure 4H; the difference increases from 2.5 to 4.2 times with the increase in curvature radius. This variation in difference is because a larger preload is required to guarantee the contact status for the mismatching curvatures, leading to more apparent geometrical distortion (especially for sticking structures). Overall, the grown structures appear to have greater adhesive force on curved or flat surfaces than on sticking structures.

The column surface is a typical developable in which the adhesive structures fabricated on the flat substrate can sometimes be stuck into the curved surface. It needs a complex tailoring or mapping design for undeveloped surfaces, such as spheres, which means a high demand for conventional fabrication. Figure 4I demonstrate the adhesive performance of the grown structure and sticking structure on a testing surface (R = 13.11 mm). The sticking structure is obtained by tailoring the adhesive structures to a series of strips and then attaching them to the spherical surface. Similarly, the adhesive force becomes more significant and constant as the preload increases. The adhesive force of the grown structure is much larger than that of the sticking structure, with a difference of 25 times at the saturation value. This difference is much larger than that of column surfaces since it is an undevelopable surface, i.e., the structural distorts the sticking structure. The influence of applied voltage on the adhesive performance of the grown structure on a curved surface is also explored for the undevelopable sphere (Figure S7, Supporting Information). The adhesive performance increases with the applied voltage, similar to that of grown structures on a flat substrate (Figure 4C). Consequently, the voltage modulation on the grown structures and adhesive performance is adequate for both flat and curved surfaces, highlighting the simplicity and university of in‐site growing strategy on target surfaces. Additionally, Figure 4J demonstrates the cyclic test of grown structures on the spherical surface. The adhesive force is kept at 5 N without sharp decline, exhibiting excellent robustness. This is crucial for promoting the grown adhesive structures in robotic manipulation applications.

## 3 Conclusion

In summary, we propose a mushroom‐shaped adhesive structure produced by an electrical in‐site growth strategy (the direct formation of a complex morphology on target surfaces from a flat film). Activated by an electrical modulation, an electrostatic force generated at the air−polymer interface drives the polymer to transform the viscous force and surface tension to move toward the upper electrode, generating the mushroom stem. Driven by the electrowetting force, the polymer spreads on the upper electrode surface when contacting it, forming a mushroom tip. Numerous polymers can be adapted to fabricate mushroom‐shaped adhesive structures of PDMS, PU, PUA, NOA, etc., due to the electrostatic force mainly determined by the material permittivity. When an electric field passes through the polymer film, the electrostatic force is produced regardless of the electrical properties and geometrical shape of the target surface. Therefore, the adhesive structures are in‐site grown on flat and curved surfaces (developable or undevelopable), including glass, metal, PET, and others. The electric field‐driven in‐site growth strategy is a universal and simple method to fabricate mushroom‐shaped structures with the structural morphology modulated by voltage, air gap, etc. Compared with the conventional approaches, there is no apparent structural deformation at the interface between the grown structures and the testing surface, corresponding to a more significant adhesive force. Generally, the difference in the adhesive strength of grown structures to sticking structures is severalfold for developable surfaces (such as columns) and up to 25 times for undevelopable surfaces (such as spheres), which traditional methods have never achieved. The in‐site grown mushroom‐shaped structures on target surfaces exhibit superior adhesion performance on numerous surfaces, including weight, beaker, and globe holder. Moreover, the adhesive force remains unchanged after 200 cycles, demonstrating remarkable reproducibility, a critical factor in robotic applications. The proposed strategy can promote the application of gecko‐inspired adhesives to a wide range of material surfaces, from flat to curved surfaces, opening an avenue for developing gecko‐inspired adhesives.

Currently, due to the limitations of the experimental conditions, the size of the adhesive structures obtained via in situ growth is difficult to be smaller than several micrometers or even at the nanoscale. In practice, nanoscale adhesive structures can be grown as long as the distance between the electrode plates and the initial polymer thickness can be precisely controlled (Figure S19, Supporting Information), which needs a sophisticated control system and would be dept studied in future work. In addition, the mushroom‐shaped structures grown on conventional curved surfaces are experimentally explored (column or spherical surfaces, for instance). It is also possible to obtain adhesive structures on more complex surfaces, such as wavy surfaces, by using an upper electrode plate that matches the morphology of the supporting substrate and controlling the uniformity of the air gap and polymer thickness (Figure S14, Supporting Information), which would be studied in the future for engineering applications.

## 4 Experimental Section

### Materials

Unless stated otherwise, solvents and chemicals were obtained commercially and used without further purification. Polydimethylsiloxane (PDMS, Sylgard 184) was obtained from Dow Corning Inc. (USA). Polyurethane (PU, Vytaflex 40) was purchased from Smooth‐On Inc. (USA). Polyurethane acrylate (PUA, UV‐Crystal Glue) was bought from Chongqing Silicon Ning Technology Co., Ltd (China). Norland Optical Adhesive (NOA 65) was supplied by Norland Products Inc. (USA). Indium tin oxide (ITO) glass and non‐conductive glass were provided by Luoyang Guluo Glass Co., Ltd (China). The conductive metal of stainless steel 304 was obtained from Ningbo Tianhui Precision Machinery Co., Ltd (China). Glass lenses with different curvatures were purchased from Shenzhen Extreme Optics Co., Ltd (China). Amorphous fluoroplastics solution (AFs) (6 wt%, AF1601) was obtained from the Chemours Company (USA), which acted as the dielectric layer on the upper electrode surface.

### Electrical Growth Process for Mushroom‐Shaped Structure on Target Surfaces

Plasma treatment of the supporting substrate was performed to enhance the bonding strength between the grown structure and the supporting substrate, thereby preventing the separation of the grown structure from the supporting substrate during the adhesion behavior. The forming material was then spin‐coated for a flat substrate to obtain the polymer film. Subsequently, PI film acting as a dielectric spacer was placed between electrode pairs to form the sandwich configuration, composed of upper electrode/air gap/polymer/lower electrode, for the subsequent electrical growth process. A Teflon film (100 nm thick) was coated on the bottom surface of the upper electrode to introduce the electrowetting effect, which is beneficial for removing the upper electrode after the growth process. The polymer was then vertically grown to the upper electrode and horizontally grown along the bottom surface of the upper electrode. In the curing process via thermal field or UV, the voltage was applied on the electrode pairs to maintain the polymeric morphology. The mushroom‐shaped structures were obtained when the upper electrode was removed. The process for growth on the curved substrate was identical except for the curved surfaces acting as the electrode pair.

### Structures and Adhesion Characterization

The microstructure of adhesive material was observed by scanning electron microscopy (SU8010, Hitachi, Japan). The adhesive force of the material was characterized by a computer servo pull–pressure test machine (PT–1176, Baoda, China). The adhesion of grown structures on flat or curved surfaces was measured by Load–Pull mode. The probe was first contacted with the sample to generate a specific contact area, and then the reverse movement was performed until complete separation was achieved. The maximum tensile force generated before separation was defined as the maximum adhesive force. The grown structures were attached to the base and adjusted to parallel the probe surface. The testing surface was moved down at a 1 mm min^−1^ speed to contact with the sample and reached a defined preload for 5 s, then moved up until the testing surface was separated from the core‐shell structures. The adhesive force was deduced from the time–force curve.

## Conflict of Interest

The authors declare no conflict of interest.

## Author Contributions

J.S. and H.T. conceived the idea. H.T., Y.L., and D.W. developed the materials for the adhesive structures. H.T., Y.L., and D.W. designed and performed the growth experiments. Y.J., T.L., X.L., and Q.C. designed and performed the adhesive performance testing. H.T. and Y.L. performed the numerical simulation of the structure growth process. H.T., D.W., C.W., and X.C. performed the numerical simulation of adhesive structure on flat/curved surfaces. J.S. supervised and directed the research. H.T., Y.L., D.W., and J.S. wrote the manuscript.

## Supporting information



Supporting Information

Supplemental Movie 1

Supplemental Movie 2

Supplemental Movie 3

## Data Availability

The data that support the findings of this study are available from the corresponding author upon reasonable request.
