# Supplementary material for: In Situ Growth of Mushroom‐Shaped Adhesive Structures on Flat/Curved Surfaces via Electrical Modulation
Source: Adv Sci (Weinh). 2024 Nov 5;11(48):2408680. doi: 10.1002/advs.202408680 (PMC11672313; doi:10.1002/advs.202408680)
Supplement: Supplementary file 1 — Supporting Information [file ADVS-11-2408680-s001.pdf]

ADVANCED SCIENCE Open Access

Supporting Information

for *Adv. Sci.*, DOI 10.1002/advs.202408680

In Situ Growth of Mushroom-Shaped Adhesive Structures on Flat/Curved Surfaces via Electrical Modulation

Hongmiao Tian, Yingze Li, Duorui Wang, Qi Chen, Yuanze Jiang, Tianci Liu, Xiangming Li, Chunhui Wang, Xiaoliang Chen and Jinyou Shao**

Supporting Information for

In situ growth of mushroom-shaped adhesive structures on flat/curved surfaces via electrical modulation

Hongmiao Tian[1], Yingze Li[1], Duorui Wang[1,2]*, Qi Chen[1], Yuanze Jiang[1], Tianci Liu[1], Xiangming Li[1,2], Chunhui Wang[1], Xiaoliang Chen[1,2] and Jinyou Shao[1,2]*

[1]Micro-and Nano-technology Research Center, State Key Laboratory for Manufacturing Systems Engineering, Xi'an Jiaotong University, Xi'an, Shaanxi 710049, China.

[2]Frontier Institute of Science and Technology (FIST), Xi'an Jiaotong University, Xi'an, Shaanxi 710049, China.

*Corresponding author. Email: duoruiwang@xjtu.edu.cn; jyshao@xjtu.edu.cn

Table of Contents

Supporting Sections

Section S1. Numerical analysis of growth process for polymer film under an electric field
Section S2. Numerical analysis of grown mushroom-shaped structures contacting to and separating from testing surface

Supporting Figures

Fig. S1. Grown mushroom shaped structures on flat substrate with forming materials of PDMS, NOA, PU and PUA.
Fig. S2. Distribution of electric field and electrostatic force on the air–polymer interface at the initial growing stage.
Fig. S3. Convex surface adopted as the target substrate for exploring the influence of applied voltage on the grown mushroom shaped structure.
Fig. S4. Curved surfaces adopted as the target substrates, composed of convex and spherical morphology, respectively.
Fig. S5. Mushroom-shaped adhesive structure fabricated on flat substrate by conventional method (photolithography and molding technique adopted here) and its corresponding adhesive force.
Fig. S6. Grown mushroom shaped structures on flat substrate with applied voltage of 200 V, 300 V, 400 V and 500 V, respectively.
Fig. S7. Influence of applied voltage on the adhesive performance of grown structure on spherical surface.
Fig. S8. Schematic of initial geometry for polymer film under an exerted electric field.
Fig. S9. Schematic of grown structure and sticking structure on curved surface
Fig. S10. Sketch of traction-separation response for cohesive behavior of adhesive structures in the numerical simulations.
Fig. S11. Numerical simulations obtained the dynamic evolution of polymer film with (A) voltage of 400 V, 600 V, 800 V and (B) air gap thickness of 5 μm, 20 μm, and 35 μm.
Fig. S12. Dynamic behavior of the grown and sticking structures on concave surfaces.
Fig. S13. Contact lines and adhesive forces as a function of time during contact- separation of grown and sticking structures on convex surfaces in finite element analysis.
Fig. S14. Finite element simulation of process of polymers growing as arrays of mushroom shaped structures on wave surfaces driven by electric fields.
Fig. S15. Finite element analysis of the separation behavior of a flat pillar from a flat surface.
Fig. S16. Stress clouds at the interface between the convex surface and grown structure and sticking structure at the moment of the inversion stage in the finite element analysis.
Fig. S17. Mushroom-type structures grown in situ at different locations on the convex surface.
Fig. S18. Microstructure arrays with different morphologies obtained by controlling the electric field intensity.
Fig. S19. Numerical simulations obtained the dynamic evolution of polymer film with air gap thickness of 100 nm.

Supporting Tables

Table S1. Parameters used in numerical simulation of growth process

Table S2. Hyperelastic models and material properties for mechanical analysis of adhesive structures

Supporting Movies

Movie S1. Numerically dynamic growing evolution of polymer film under an external electric field for curved substrate.

Movie S2. Numerically dynamic contacting–separating behavior of grown structure on a convex surface.

Movie S3. Numerically dynamic contacting–separating behavior of sticking structure on a convex surface.

Supporting Text

Section S1. Numerical analysis of growth process for polymer film under an electric field

The Cahn–Hilliard model, which assumes that the interface thickness between two phases in a system is small but greater than the actual physical one, was used to represent the two immiscible components, namely air and ploymer film. A smooth function φ, called as the order parameter, is used to geometrically define one phase. This function is equal to 1 in the ploymer phase and -1 in the air phase, and it varies continuously in the interfaces between the polymer phase and the air phase. From a mathematical point of view, this approach may be understood as a regularization of sharp interface models with small interfacial thickness.

The dual-phase of air and fluidic polymers can be described via ternary Cahn–Hilliard equation, as follows:

$$\begin{cases} \dfrac{\partial \varphi}{\partial t}+\nabla\cdot(u\ \varphi)=\nabla\cdot(M_0\nabla G) \\ G=\dfrac{3\sqrt{2}}{4}\gamma\left[-\chi\nabla^2\varphi+\dfrac{\varphi(\varphi^2\text{-}1)}{\chi}\right] \end{cases} \tag{1}$$

wherein φ is the phase function (i.e., order parameter), u is the the fluid velocity, M is interface mobility (also depended on φ), G is chemical potential, γ is the Air–polymer interface surface tension coefficient, χ is the interfacial thickness.

The Navier–Stokes equations of momentum and mass conservation for the motion of the fluid are written as

$$\begin{cases} \rho\dfrac{\partial u}{\partial t}+\rho u\cdot\nabla u=-\nabla P+\eta\nabla^2 u+\dfrac{1}{3}\eta\nabla(\nabla\cdot u)+f_e+F_{st}+\rho g \\ \dfrac{\partial(\rho u)}{\partial t}+\nabla\cdot(\rho u)=0 \end{cases} \tag{2}$$

where the density ρ and viscosity η are smooth functions that depend on the order parameters, P is the fluidic pressure, f_e denotes the volumetric electrostatic force generated by an electric field, g is

the gravitational acceleration, and F is the surface tension at the air–polymer interface. Here, the polymer can be considered as an incompressible and isotropic Newtonian fluid.

In the growth process, the fluid can be considered as the pure dielectric polymer; thus, the conductivity of fluids can be ignored in the numerical simulations. Here, the distribution of electric field inside the numerical domain can be depicted as follows:

$$\nabla\cdot(\varepsilon E)=0 \quad \varepsilon=\varepsilon_0\varepsilon_r \tag{3}$$

wherein ε is the permittivity of fluids, ε_0 is the permittivity of vacuum, ε_r is the relative permittivity of air and polymer, and E is the electric field in the numerical domain ($E=-\nabla V$ with V representing electric potential).

Once an external electric field is applied on the fluids, the Maxwell stress tensor would be generated in the air and polymer with the expression of

$$T^e=\varepsilon EE - \frac{1}{2}E^2\left[\varepsilon - \rho\left(\frac{\partial\varepsilon}{\partial\rho}\right)\right]I \tag{4}$$

with I is the unit stress tensor. According to the Maxwell stress tensor, we can obtain the electrostatic force acting on the fluids, by

$$f_e=\nabla\cdot T^e=\rho_f E - \frac{1}{2}E^2\nabla\varepsilon + \nabla\left(\frac{1}{2}E^2\rho\frac{\partial\varepsilon}{\partial\rho}\right) \tag{5}$$

where ρ_f is the space charge. Here, the experimental materials are considered as pure dielectric, i.e., no space charge, and the relative permittivity of material is not depended on the position. Thus, the expression of electrostatic force can be expressed as

$$f_e=-\frac{1}{2}E^2\nabla\varepsilon \tag{6}$$

It can be seen that the electrostatic force of f_e is concentrated on the interface, and would become nearly zero as the position away from the interface. This can be attributed to the gradient of permittivity of used materials.

Under dielectric wetting effect, we can obtain the electrowetting contact angle by

$$\cos(\theta) = \cos(\theta_0) + \frac{\varepsilon_r U^2}{2\gamma_{lv} d} \tag{7}$$

where θ is the electrowetting contact angle, θ_0 is the natural contact angle, U is the applied voltage, ε_r is the dielectric constant of the dielectric layer, γ_{lv} is the surface tension coefficient at the polymer-air contact surface, and d is the thickness of the dielectric layer.

In order to better simulate the dynamic evolution process of contact Angle in polymer rheological behavior in numerical analysis, the connection between electrowetting angle and phase function can be established as:

$$n \cdot \nabla\varphi = \cos(\theta)\,|\nabla\varphi| \tag{8}$$

with n standing for the unit normal vector of the solid surface.

Considering the difference between the rheological behavior of polymer fluid at micro and nano scale and that at macro scale, the dynamic viscosity coefficient of polymer fluid at micro and nano scale can be obtained via Eringen theory, by:

$$\eta = \eta_0 \left[1 + \zeta\left(\frac{r}{L}\right)^2\right] \tag{9}$$

with η standing for dynamic viscosity coefficient of polymer fluid at microscale, η_0 for dynamic viscosity coefficient of polymer fluid at macroscopic scale, ζ for material property parameter, r for turning radius and L for the distance between a polymer molecule and a solid surface.

The iteration of this coupled problem is carried out by the finite element method in commercial software of COMSOL Multiphysics, in which the governing equations described in the preceding are defined explicitly and symbolically and then automatically solved by the Galerkin approach. Once the order parameter φ is obtained, the moving liquid−liquid interface can be easily identified by simply color–mapping the order parameter, providing a visualization of the bilayer film deformation

step by step.

The relevant simulation parameters for both flat and prepatterned bilayer film are shown in Supporting Table 1.

Section S2. Numerical analysis of grown mushroom-shaped structures contacting to and separating from testing surface.

To mimic the contact behavior of an adhesive structure on rough surface, cohesive surface model was employed. The sketch of grown structure and sticking structure on curved surface is shown in Supporting Fig. 9. The curvature of the curved surface and the target surface is consistent with the reality. In order to simplify the workload, the number of contact adhesive structures was reduced in the analysis, and the size of individual adhesion structures is scaled up in equal proportions, thus compensating for the lack of comparison of numerical analysis results caused by the reduction in the number of contact structures. The analysis defaults to the fact that the two adhesive structures are well combined with the target substrate, and only considers the effect of changes in cap rim morphology due to the different combine modes of the adhesion structures to the substrate.

Considering the much larger modulus of ground substrate (i.e., rough surface) in comparison with adhesive structure, the ground substrate was considered as rigid body during simulations, and the adhesive structure was set as deformed body. For the adhesive structure, material properties of PDMS were adopted as the structural material, which is usually used to fabricate adhesive structures according to the published literatures. the hyperelastic tensile behavior of polymeric materials were described by Neo–Hookean model, with the model parameters for different materials given in Supporting Table 2.

In the downpressing phase, we use general contact to simulate contact, and in the pull-up phase, we introduce cohesive contact property. The formula of the cohesive constitutive law is as follows:

$$F=\begin{Bmatrix} F_n \\ F_s \\ F_t \end{Bmatrix}=A\begin{bmatrix} K_{nn} & K_{ns} & K_{nt} \\ K_{sn} & K_{ss} & K_{st} \\ K_{tn} & K_{ts} & K_{tt} \end{bmatrix}\begin{Bmatrix} \delta_n \\ \delta_s \\ \delta_t \end{Bmatrix}=AK\delta \qquad (10)$$

Among them, F_n represents the normal separation force on the interface during the separation

process, F_s and F_t are two shear separation forces. δ_n、δ_s、δ_t represent the opening displacement of the cohesive interface in three directions, A represents the total area of the interface; K represents the interface stiffness matrix.

In the numerical simulation of the contact and separation process, the adhesive layer and substrate are fully bound, and the bottom of the substrate is fully immobilized. In the actual test, the test object first moved downward at a constant speed to contact the adhesive layer, and then moved upward at a constant speed until it was completely separated, so a constant displacement speed along the Y direction was applied on the upper surface of the concave probe.

In the ABAQUS/Standard contact analysis, the contact pair is often composed of the master surface and the slave surface, and the contact direction is always the normal direction of the master surface. Since the stiffness of the adhesive film is relatively small relative to the test probe, the surface of the concave probe was selected as the main surface, and the surface of the adhesive layer was the secondary surface. In addition, the cohesive contact is selected when defining the contact in the separation analysis step, the criterion of interface fracture damage is the maximum nominal stress, the specific parameters are 0.45, 0.45, 0.45, the interface stiffness is 5000 N/mm, and the fracture energy is 1×10^{-5} mJ.

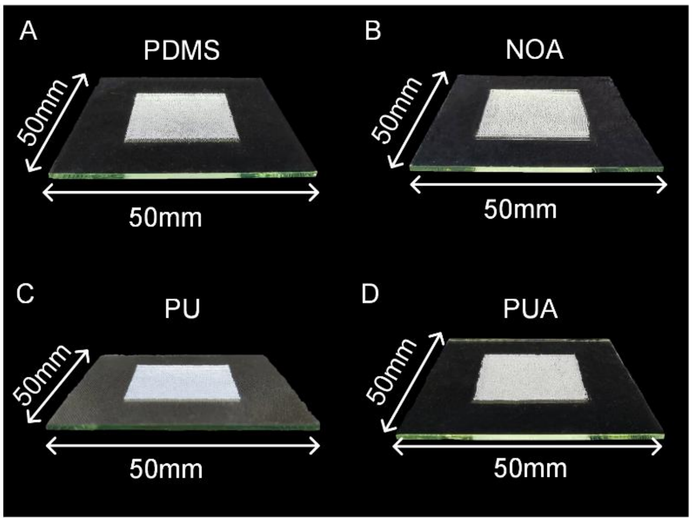

Supporting Fig. 1 | Grown mushroom shaped structures on flat substrate with different forming materials of (A) polydimethylsiloxane (PDMS), (B) Norland optical adhesive (NOA), (C) polyurethane (PU) and (D) polyurethane acrylate (PUA).

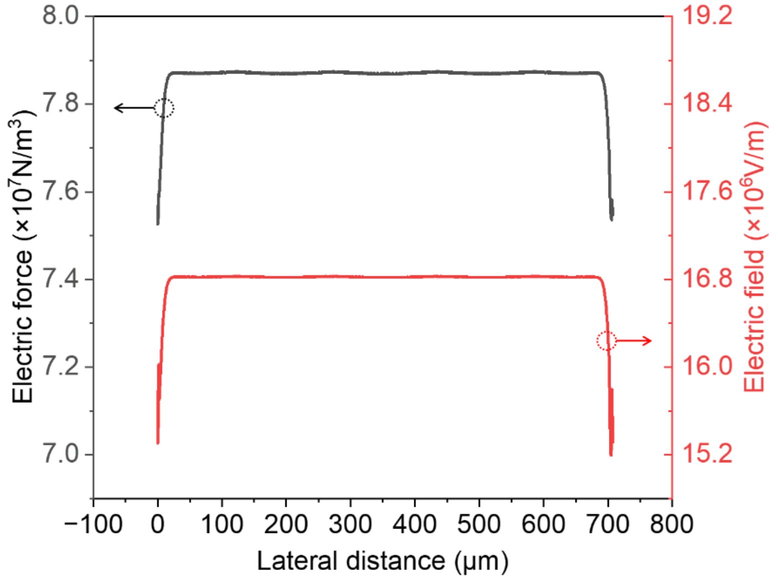

Supporting Fig. 2 | Distribution of electric field and electrostatic force on the air–polymer interface at the initial growing stage. Obviously, the distribution of electrostatic force is similar to that of electric field, implying that the electrostatic force acting on the bilayer film is determined by the electric field.

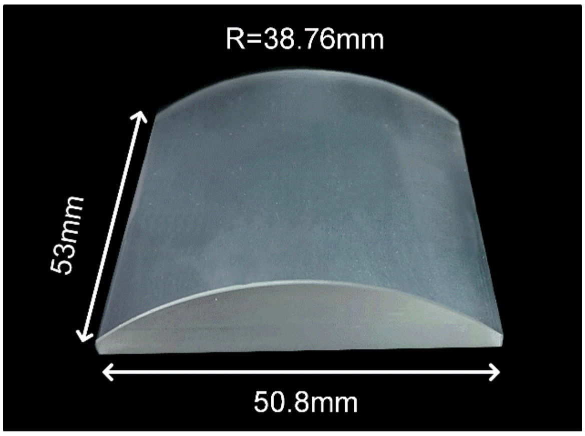

Supporting Fig. 3 | Convex surface adopted as the target substrate for exploring the influence of applied voltage on the grown mushroom shaped structure.

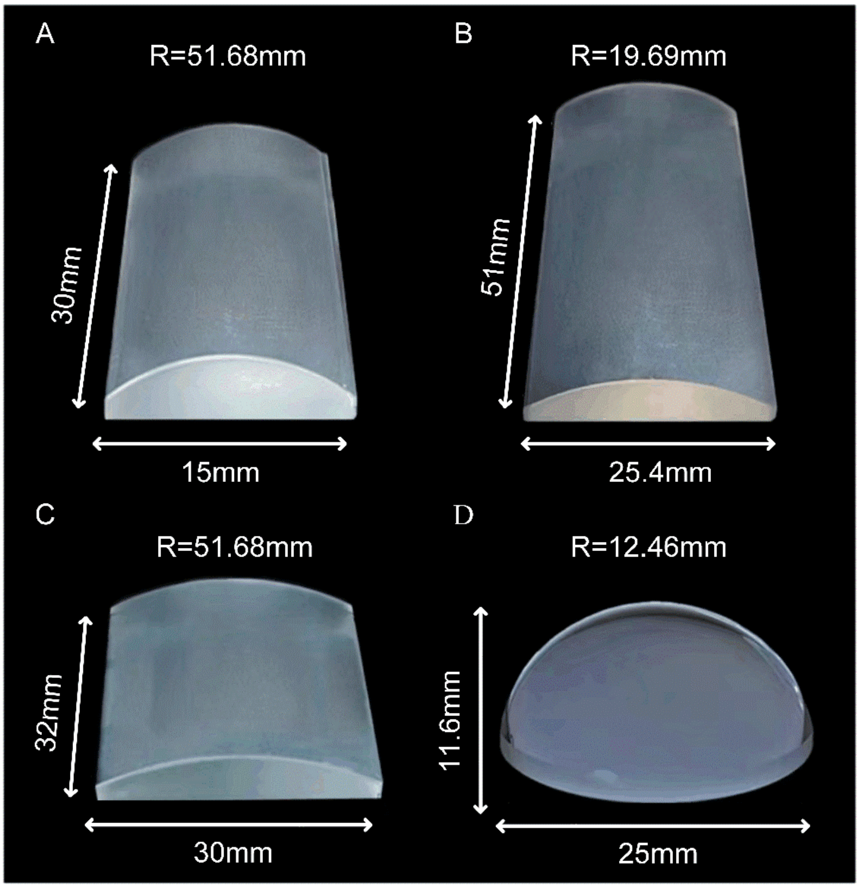

Supporting Fig. 4 | Curved surfaces adopted as the target substrates, composed of convex and spherical morphology, respectively. (A)~(C) are convex surfaces with radius of curvature 10.34 mm, 19.69 mm and 51.68 mm, respectively, and (D) is an undevelopable spherical surface with radius of curvature 12.46 mm.

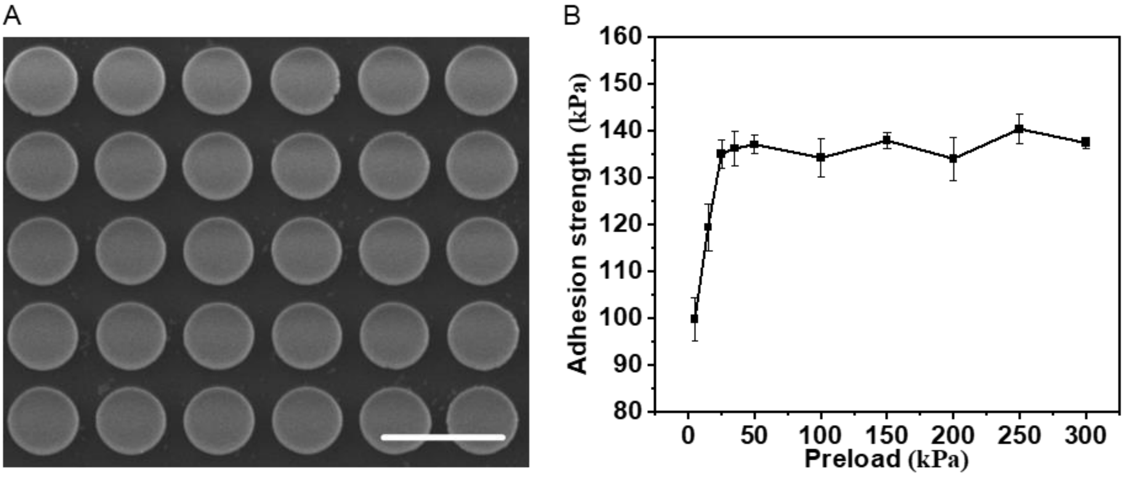

Supporting Fig. 5 | (A) Mushroom-shaped adhesive structure fabricated on flat substrate by conventional method (photolithography and molding technique adopted here) and (B) its corresponding adhesive strength. It can be seen that on flat surfaces, the mushroom structures fabricated using the in situ growth method proposed herein have similar adhesion strength as those fabricated using conventional methods. The scale bar is 50 μm.

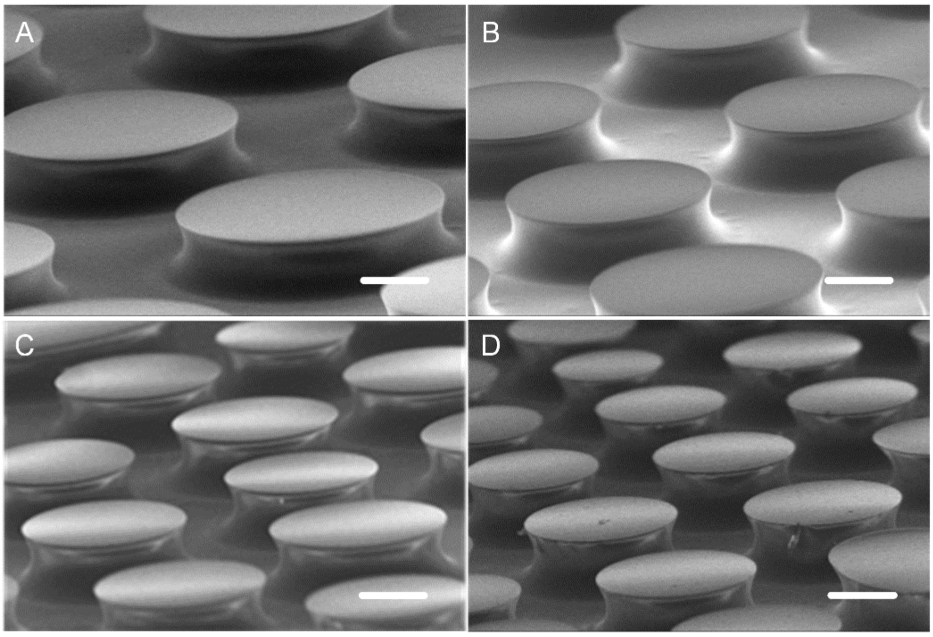

Supporting Fig. 6 | Grown mushroom shaped structures on flat substrate with applied voltage of (A) 200 V, (B) 300 V, (C) 400 V and (D) 500 V, respectively. The effect of the applied voltage on the morphology and distribution period of the mushroom structures grown on the flat surface can be observed. The scale bar is 100 μm.

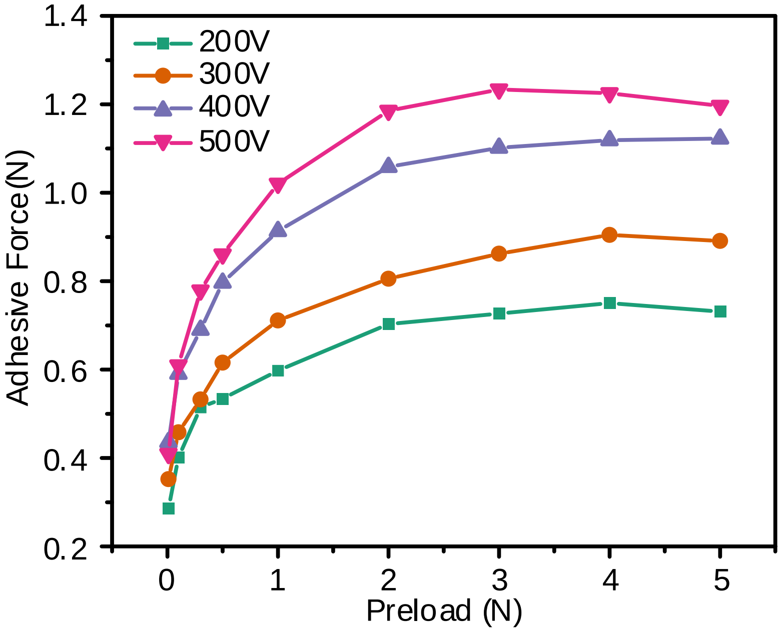

Supporting Fig. 7 | Influence of applied voltage on the adhesive performance of grown structure on spherical surface. Obviously, the adhesive force of the adhesive structures on the spherical surface can be modulated by varying the applied voltage.

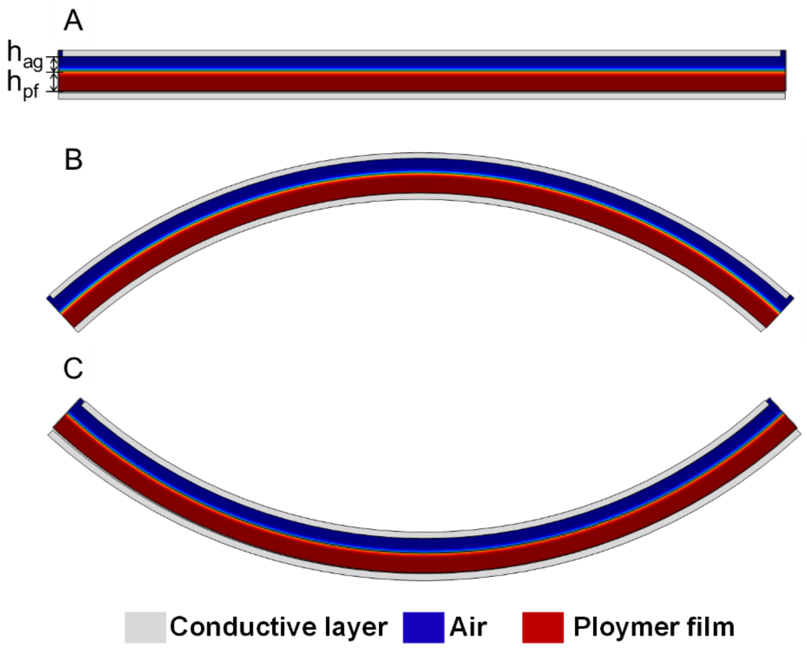

Supporting Fig. 8 | Schematic of initial geometry for polymer film under an exerted electric field on (A) flat surface, (B) convex surface and (C) concaved surface, where h_{ag} and h_{pf} represent the thickness of the air gap and the thickness of the polymer film, respectively.

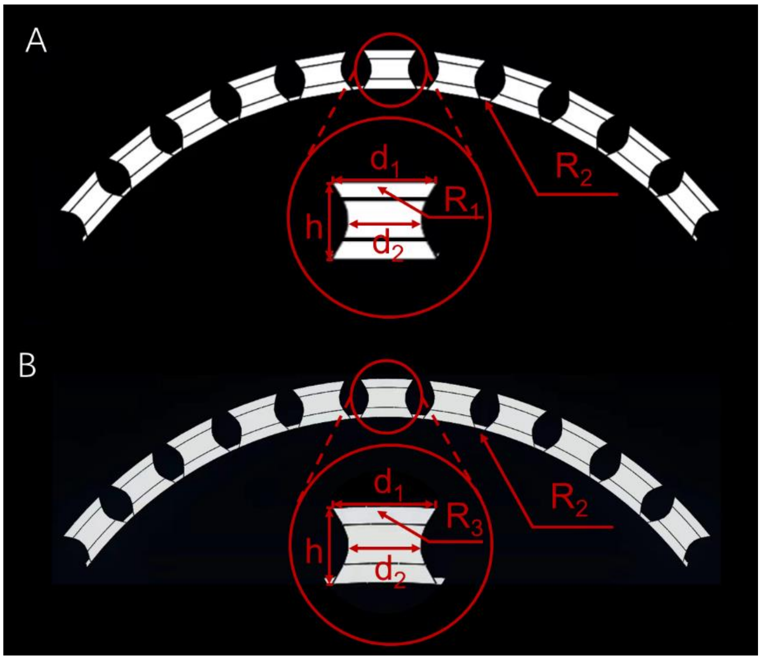

Supporting Fig. 9 | Schematic of sticking structure (A) and grown structure (B) on curved surface. The difference between the two is that the radius of curvature R_1 at the top of the pasted structure is defined to be infinity, while the radius of curvature R_3 at the top of the growing structure is constant.

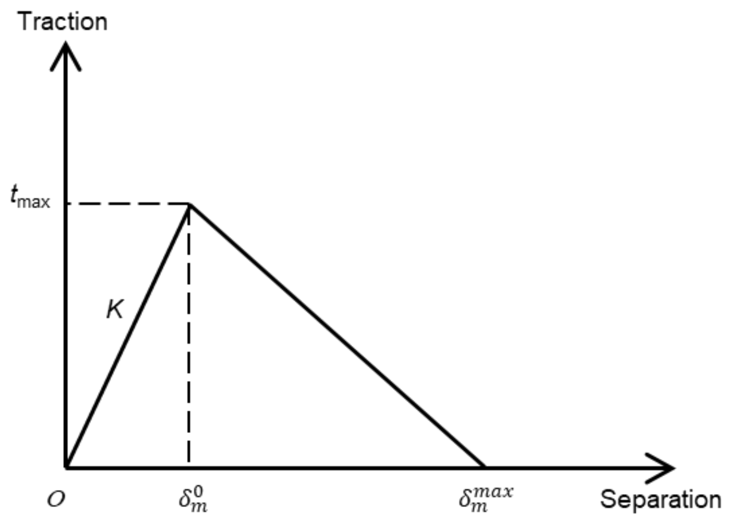

Supporting Fig. 10 | Sketch of traction-separation response for cohesive behavior of adhesive structures in the numerical simulations.

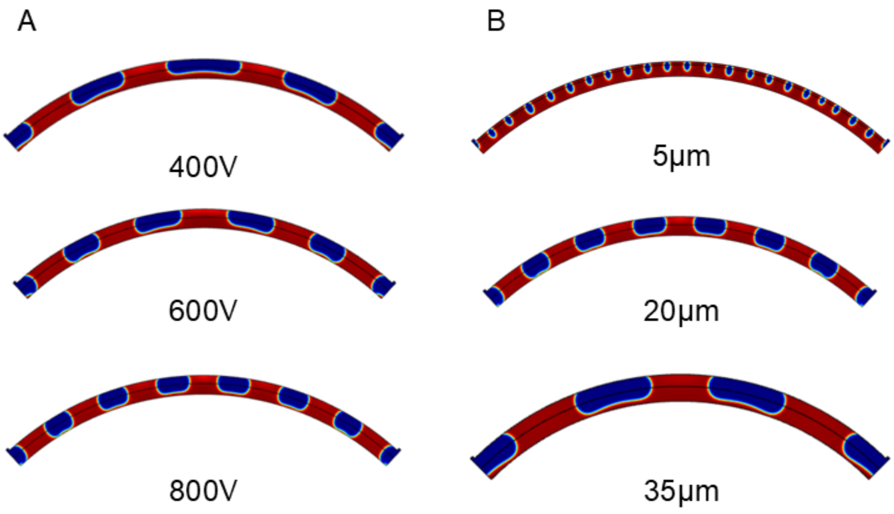

Supporting Fig. 11 | Numerical simulations obtained the dynamic evolution of polymer film with (A) voltage of 400 V, 600 V, 800 V and (B) air gap thickness of 5 μm, 20 μm, and 35 μm. Here, blue and red colors represent air and polymer, respectively. It can be observed that the size and period of the grown structures become smaller as the voltage is increased and the thickness of the air gap is decreased, respectively, which demonstrates the possibility of modulating the grown structures by adjusting experimental parameters through in situ growth strategies.

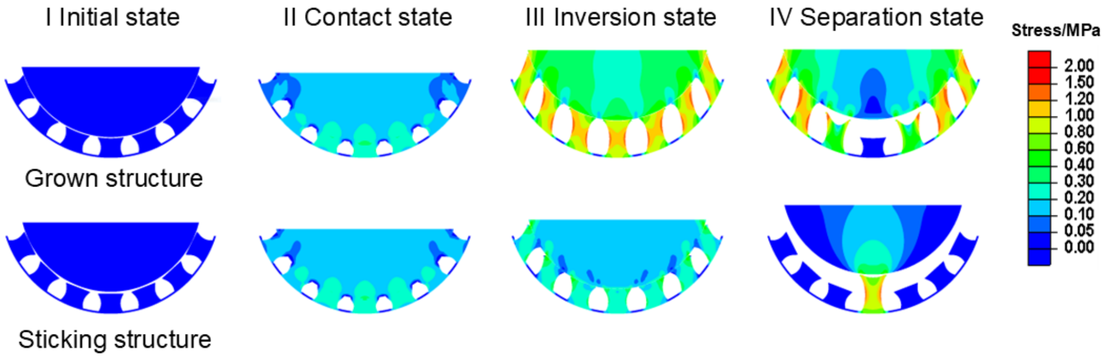

Supporting Fig. 12 | Dynamic behavior of the grown and sticking structures on concave surfaces at (I) initial state, (II) contact stage, (III) inversion state, and (IV) separation state; cloud atlas representing the internal stress. Similar to the contact-detachment behavior of adhesive structures grown/sticked on convex surfaces, the interfacial stress concentration of the sticking structure is more pronounced than that of the grown structure in the contact and inversion states.

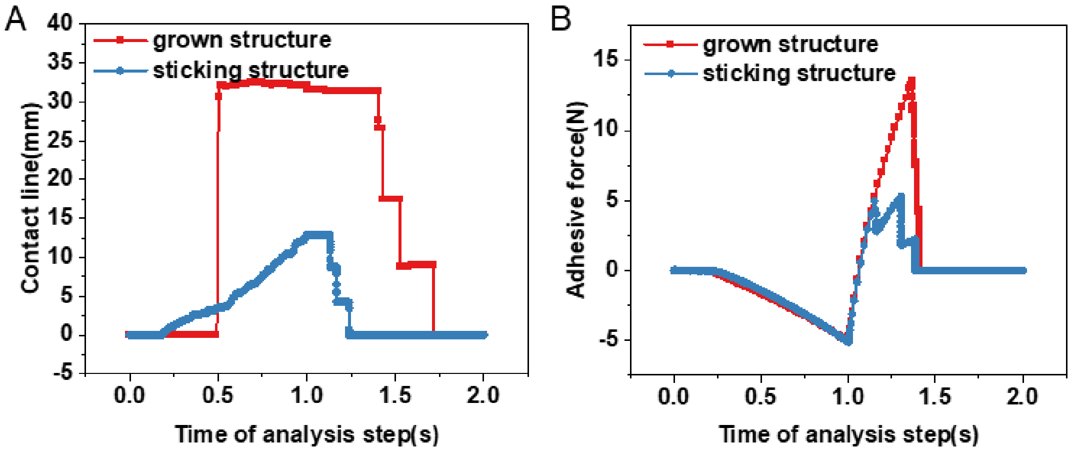

Supporting Fig. 13 | Contact lines and adhesive forces as a function of time during contact-separation of grown and sticking structures on convex surfaces in finite element analysis. The grown structures are more likely to be in conformal contact with the convex surface and difficult to separate from it, and thus their contact area is always significantly larger than that of the sticking structures, leading to greater adhesion in the separated state.

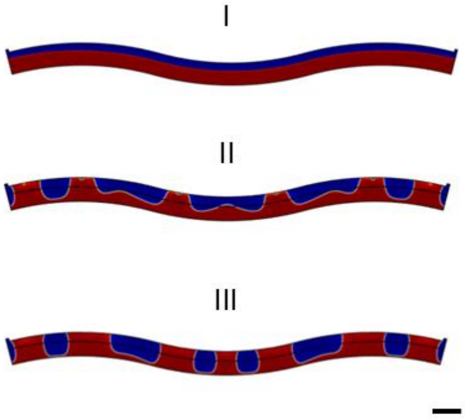

Supporting Fig. 14 | Finite element simulation of process of polymers growing as arrays of mushroom shaped structures on wave surfaces driven by electric fields. Here, blue and red colors represent air and polymer, respectively. Stages I, II, III are the initial stage, vertical growth stage and horizontal growth stage respectively. The scale bar is 20 μm.

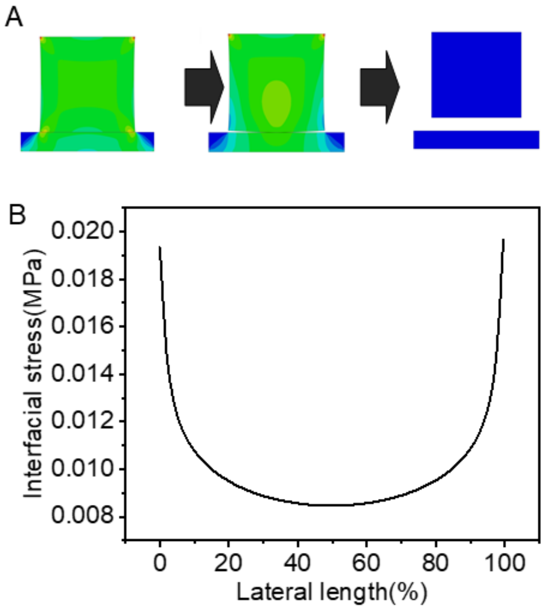

Supporting Fig. 15 | Finite element analysis of the separation behavior of a flat pillar from a flat surface. (A) Diagram of the process of separating a flat pillar from a flat surface. (B) Interfacial stresses at the initial moment of the separation process. The maximum stress between interfaces is concentrated at the edges of the pillar.

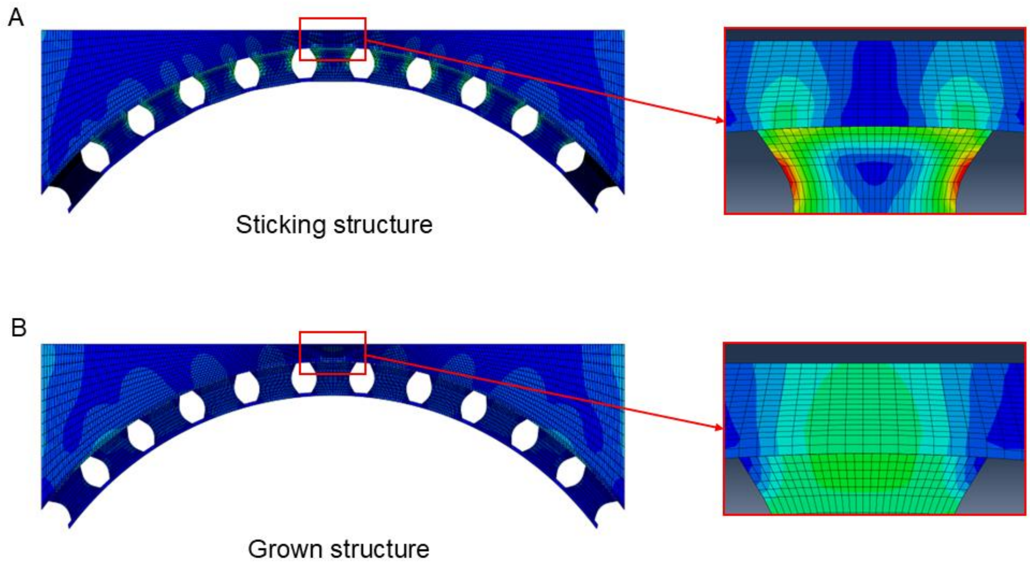

Supporting Fig. 16 | Stress clouds at the interface between the convex surface and (A) grown structure and (B) sticking structure at the moment of the inversion stage (Stage III) in the finite element analysis. Since the curvature of the surface of the sticking structure is larger than that of the concave surface, the interfacial stress between them is concentrated at the edge of the sticking structure, while the interfacial stress between the grown structure and the concave surface is uniformly distributed.

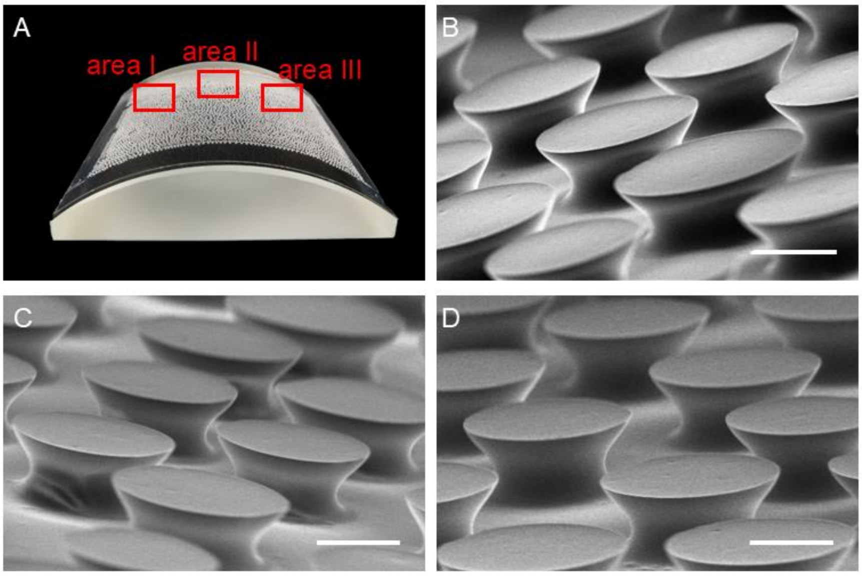

Supporting Fig. 17 | Mushroom-type structures grown in situ at different locations on the convex surface. (A) Convex surface with mushroom-shaped structures grown on it and mushroom structures grown on (B) area I, (C) area II and (D)area III, respectively. It can be observed that the mushroom-shaped structures in different areas have almost the same size and period. The scale bars are 400 μm.

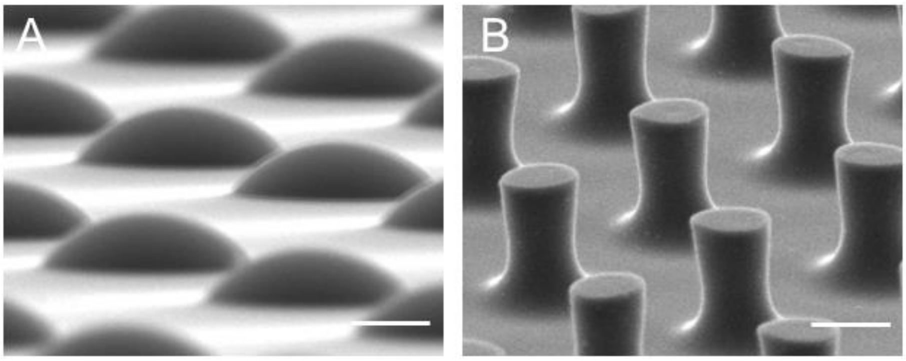

Supporting Fig. S18 | Microstructure arrays with different morphologies obtained by controlling the electric field intensity. (A) The grown micro-lens obtained with a large distance between the electrodes. (B) The grown pillars obtained without the dielectric layer. The large distance between the electrodes in (A) result in low electrostatic force (corresponding to the electric field intensity) which is not sufficient to drive the polymer to conquer viscous force and surface tension to move upwards, thus the micro-lens are obtained. The electrostatic force during the growth process in (B) cannot drive the polymer to move along the bottom surface of the upper electrode after contacting each other, so the mushroom cap cannot be formed and the pillars are obtained. The scale bars are 50 μm.

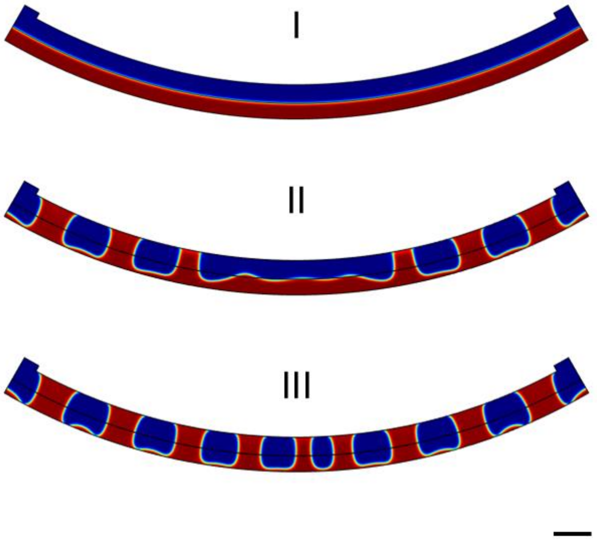

Supporting Fig. S19 | Numerical simulations obtained the dynamic evolution of polymer film with air gap thickness of 100 nm. Here, blue and red colors represent air and polymer, respectively. Stages I, II, III are the initial stage, vertical growth stage and horizontal growth stage respectively. It can be observed that mushroom-shaped structures in tens of nanometer are obtained on the curved surface, which demonstrates the possibility of fabricating mushroom-shaped structures in nanoscale on curved surfaces through in situ growth strategy. The scale bar is 100 nm.

Supporting Table S1. Parameters used in numerical simulation of growth process

Simulation parameters	Value
Air–polymer interface surface tension coefficient	0.03 N/m
Air viscosity	1.79×10^{-5}
Polymer viscosity	2 Pa • s
Air mass density	1.29 kg/m^3
Polymer density	1000 kg/m^3
Air relative permittivity	1
Polymer relative permittivity	4
Voltage	400 V
Air gap	15 μm
Polymer thickness	20 μm

Supporting Table S2. Hyperelastic models and material properties for mechanical analysis of adhesive structures

Materials	Models	Parameters
Silicone rubber	Neo-Hooke	C_{10}=0.17，D_1=0.24
Glass	Elasticity	E=72GPa，μ=0.25
